# Electrospun-Based Membranes as a Key Tool to Prevent Respiratory Infections

**DOI:** 10.3390/polym14183787

**Published:** 2022-09-10

**Authors:** Sara F. C. Guerreiro, Carolina A. M. Ferreira, Joana F. A. Valente, Tatiana M. F. Patrício, Nuno M. F. Alves, Juliana R. Dias

**Affiliations:** 1 Centre for Rapid and Sustainable Product Development (CDRSP), Instituto Politécnico de Leiria, 2030-028 Marinha Grande, Portugal; 2 Instituto de Investigação e Inovação em Saúde (i3S), Universidade do Porto, Rua Alfredo Allen 208, 4200-135 Porto, Portugal; 3 Medical Physics Department, Portuguese Institute of Oncology (IPO-Porto), 4200-072 Porto, Portugal; 4 Abel Salazar Institute of Biomedical Sciences (ICBAS), University of Porto, Rua de Jorge Viterbo Ferreira, 228, 4050-313 Porto, Portugal; 5 Centro de Estudos de Ciência Animal (CECA), Instituto de Ciências, Tecnologias e Agroambiente (ICETA), Universidade do Porto, Praça Gomes Teixeira, Apartado 55142, 4051-401 Porto, Portugal; 6 Marine and Environmental Sciences Centre (MARE), ESTM, Instituto Politécnico de Leiria, 2050-641 Peniche, Portugal

**Keywords:** electrospun meshes, face mask filters, antimicrobial materials, bioactive materials, respiratory disease

## Abstract

The use of electrospun meshes has been proposed as highly efficient protective equipment to prevent respiratory infections. Those infections can result from the activity of micro-organisms and other small dust particles, such as those resulting from air pollution, that impair the respiratory tract, induce cellular damage and compromise breathing capacity. Therefore, electrospun meshes can contribute to promoting air-breathing quality and controlling the spread of such epidemic-disrupting agents due to their intrinsic characteristics, namely, low pore size, and high porosity and surface area. In this review, the mechanisms behind the pathogenesis of several stressors of the respiratory system are covered as well as the strategies adopted to inhibit their action. The main goal is to discuss the performance of antimicrobial electrospun nanofibers by comparing the results already reported in the literature. Further, the main aspects of the certification of filtering systems are highlighted, and the expected technology developments in the industry are also discussed.

## 1. Introduction

Lower respiratory infections cause more than 4 million deaths annually worldwide, resulting in a high implication on health-care costs with an estimated annual charge of more than EUR 67 billion [1,2]. In 2019, trachea, bronchus and lung cancers reported more than 1.8 million lives lost worldwide [1,3]. Generally, the high incidence, prevalence and mortality of respiratory-related pathologies show the extent of a current problem that has been exacerbated by numerous factors, including the deterioration of air quality, poor healthcare services, lack of personal protection, and the emergence of new strains that contribute to the high resistance of micro-organisms [4]. Recently, COVID-19, caused by SARS-CoV-2 virus, was an example of how a respiratory infectious disease can have a severe impact on public health and on the economy. Common respiratory complications associated with the respiratory tract are caused by the deposition of pathogens in the epithelium, resulting in damage to sensitive tissues and loss of their biological activity, often leading to permanent issues or the death of the patient [4]. Micro-organisms are not the only ones to interact directly with cells; inorganic small particles can also promote the disruption of cellular structures causing extended damage. In fact, respiratory occupational diseases resulting from work activities with exhaustive exposure to silica, asbestos or other microparticles are also considered critical and can cause serious damage to the respiratory tract [5,6]. Furthermore, exposure to smoke, volatile organic compounds and other air pollutants were identified as triggers for respiratory complications [7]. The reduction of pollutant-gases emissions and strict rules for the use of personal protective equipment are some of the principles that have been adopted to promote breathing air quality [8]. Beyond traditional techniques to produce the nonwoven fabric used in personal protective equipment, electrospinning emerged as a promising technique for its fabrication. This manufacturing technique uses polymeric solutions exposed to high voltages to obtain fibers whose diameter can range from a micrometer to a few nanometers to produce nonwoven fabric [9,10,11]. The reduced dimensions of fibrillar structures create an electrospun membrane with unique characteristics such as high porosity and narrow porous size. These structural advantages have been reported as extremely effective in the filtration of fine particulate matters (PMs), pathogenic aerosols, and volatile organic compounds (VOCs) that would otherwise be inhaled [12]. However, depending on the porosity and thickness of electrospun nanofibers, breathing capacity may be compromised. For this reason, it is challenging to ensure adequate filtration function without compromising breathing capacity.

Beyond mesh’s porosity, other characteristics of the materials used to produce the electrospun nanofibers can contribute to improving the filtration efficacy. The hydrophobicity of some electrospinnable polymeric materials proves to be useful in the filtration of aerosol that comes from coughing or sneezing and that can remain suspended in the air for hours. In fact, another advantage of electrospun meshes is the possibility to produce (i) bioactive fibers through the incorporation of biopolymers, inorganic nanoparticles (NPs) and biomolecules, and (ii) hybrid structures combining different materials and/or different deposition strategies.

Considering an economic perspective, electrospinning-derived products are extremely efficient due to the high surface–volume ratio which means low volumes of polymeric solution are used to manufacture membranes that cover a large surface area [13]. Additionally, electrospun products can also be competitive, due to the simplicity and low initial investment required to assemble a conventional electrospinning setup [14].

## 2. Action Mechanisms of Pathogenic Agents

**Figure 1:**
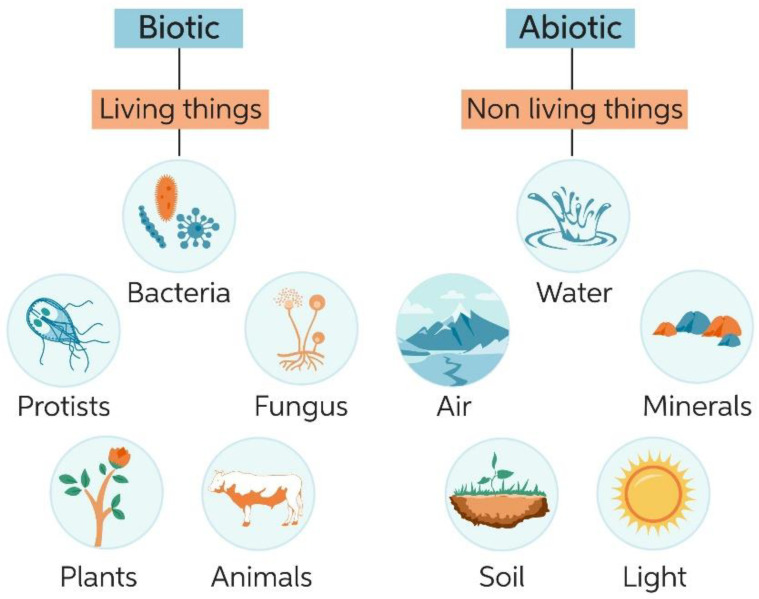
Biotic versus abiotic agents. Reproduced with permission from [21].

Viruses and bacteria can be digested by phagocytes and other defensive cells from the immune system that are responsible for their inactivation [15]. The immediate response given by defensive cells is an innate and undifferentiated mechanism that is inherited by each individual and is active from the moment of birth [16]. Additionally, an adaptative response can also be developed after previous exposure to some micro-organisms [17], after which the immune system can recognize the pathogen recruiting B-cell lymphocytes to synthesize antibodies, promoting the phagocytes’ activity and releasing inflammation mediators [18]. Moreover, multiple external factors, including other medical conditions or medication, can suppress the immune system [19,20]. Thus, the use of protective equipment against pathogens plays an imperative role for immunocompromised individuals, healthcare professionals and all citizens in epidemic scenarios. The understanding of a pathogen’s action mechanisms including abiotic and biotic agents (Figure 1), as well as their intrinsic characteristics that contribute to their spread, are crucial for the development of effective personal protective equipment. In fact, novel protective equipment development must offer a functional barrier system that combines reactive surfaces that lead to the inactivation of pathogens based on the disruption of their action mechanisms.

### 2.1. Abiotic Agents

The deposition of inorganic small particles such as silica, asbestos or coal elements in the epithelium cells is the precursor of an inflammation process that typically affects the lower respiratory tract [5,6]. Extended exposure to the air contaminated by inorganic small particles can lead to the development of respiratory-related pathologies such as silicosis or asbestosis [22]. In this condition, the inhaled and accumulated particles are ingested by macrophages to be removed from the body. Additionally, macrophages are bonded with lysosomes which contain enzymes and other molecules responsible for digesting the hazard particles [23]. However, some of these particles can induce damage to the lysosomal membrane leading to its rupture and triggering an inflammatory process [24]. This process is characterized by the release of cytokines which mediate the process by stimulating the fibroblasts to synthesize collagen to remodel the extracellular matrix [25]. However, the excessive activity of fibroblasts leveraged by severe exposures to pathogenic agents results in fibrosis due to the high accumulation of fibrillar collagen in the tissues [26]. In fact, damage resulting from long-term exposure to silica or asbestos dust is irreversible; the lungs lose their ability to stretch and respiratory capacity is affected [5,6]. 

Other abiotic agents such as chemical gases released from industrial pollution, including nitrogen dioxide, sulfur dioxide and ammonia, can also lead to tissue inflammation. For solid inorganic particles, the inflammation site is strongly dependent on particle size [7]; however, chemical gases can cause inflammation in different sites of the respiratory tract dependent on their solubility [27]. For less-water-soluble gases, they will be deeply inhaled and reach the lungs. In this situation, immune response and fibrotic changes similar to those from solid inorganic particles are observed. 

### 2.2. Biotic Agents

#### 2.2.1. Bacteria

**Figure 2:**
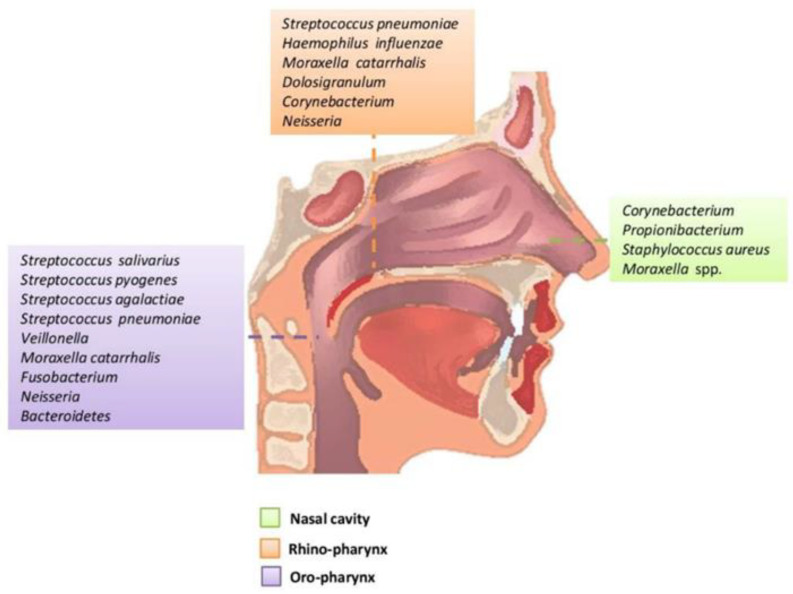
Principal genera of bacteria present in upper respiratory tract of adults. Reproduced with permission from [30].

Biotic agents are also precursors of several respiratory diseases and many of these agents are part of the human natural microbiome as bacteria [28]. The respiratory tract is colonized by a diversity of bacteria, which is strengthened from birth to adulthood, depending on some extrinsic (birth mode or feeding type) and intrinsic (genetics and epigenetics) factors [28]. The respiratory tract microbiome is composed mainly by *Genera’s Staphylococcus*, *Streptococcus*, *Corynebacterium*, *Prevotella*, *Veillonella*, *Propionibacterium*, *Dolosigranulum*, *Fusobacterium*, *Haemophilus*, and *Moraxella*, among others [29,30] (Figure 2). These organisms establish specific niches and complex symbiotic relationships, making them responsible for maintaining respiratory health [28]. However, not all the bacteria are helpful and the most common respiratory diseases are caused by commensal bacteria that change for pathogenic bacteria or from viral–bacterial interactions that arise when the equilibrium of host–microbiome or health is compromised [28]. The mechanisms used by bacteria to cause respiratory diseases are based on their inter- and intra-relationships: (i) through positive associations such as mutualism, commensalism, symbiosis, or by helping to evade the host immune system, or (ii) by negative associations by amensalism or predation, in which organisms directly compete for the same niche, or when host immune responses disproportionally affect one of the competing micro-organisms [29].

The Spanish flu is an example of that, basically: the influenza A virus has been shown to disrupt the airway–epithelial barrier, known as the first line of defense, enabling the colonization of *Streptococcus pneumoniae* (the pneumococcus) and changing the immune system response, causing a reduction in mucociliary clearance, which is a mechanism of the removal of dangerous particles and gases in the mucus from the respiratory tract [31]. Equally, Legionnaires’ disease is caused by the Gram-negative bacterium *Legionella* that can cause pneumonia. This bacterium acts by modifying the innate systems of epithelial cells and pulmonary cells overproducing proinflammatory cytokines, leading to severe inflammation [32]. Another mechanism associated with host–bacterial interactions is the production of hydrogen peroxidase by some tolerant organisms in high lethal concentrations for most of the bacteria. While other species attack the epithelium adherence structure of the competing micro-organism, as in the case of the pneumococcus that expresses neuraminidase able to cut off the cell-surface-expressed sialic acids of some *Haemophilus influenzae* (*H. influenzae*) strains, thereby preventing attachment to the surface of airway cells and subsequent colonization [29]. In-vitro studies show that another mechanism is through a beneficial host immune-system response over another species. For instance, H. influenzae, via phosphorylcholine (a cell-surface molecule that mediates bacterial adherence to host cell receptors) is benefited over Staphylococcus pneumoniae, since H. influenzae do not need phosphorylcholine to survive, therefore, pre-exposure to one of the two species induces the production of antibodies against phosphorylcholine, thus promoting the clearance of the other co-colonizing species [29]. Bacteria through siderophores can also damage the host immune system by competing for iron, which is bound to hemoglobin, transferrin, and lactoferrin. Since free iron is scarce in tissue fluids and blood, bacterial siderophores compete effectively for iron Fe^3+^ bound to lactoferrin and transferrin, and use it for growing, while preventing those proteins from being used by healthy cells [33].

Subsequently, based on these mechanisms, bacteria assure effective colonization, growth and can release pathogenic factors such as capsules, endotoxins and exotoxins that cause inflammation and diseases such as diphtheria, SARS (severe acute respiratory syndrome), asthma, tuberculosis, and otitis, among others [34]. Considering that some of these pathogenic bacteria are transmissible by aerosols, air purification is required to control their spread.

#### 2.2.2. Virus

**Figure 3:**
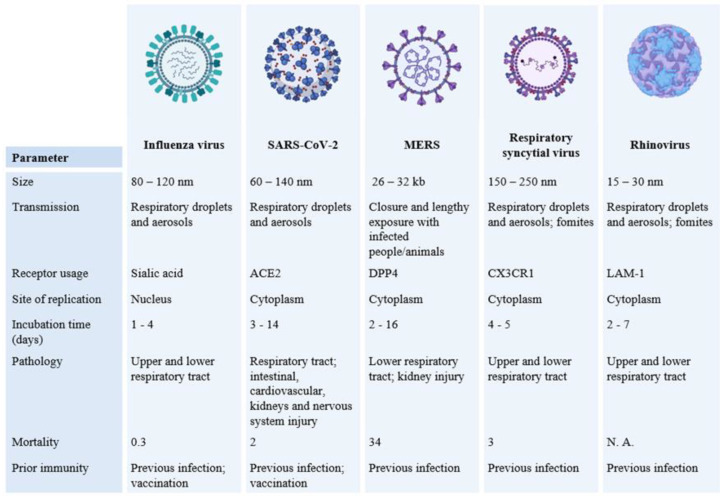
Microbiologic and epidemiologic comparisons between influenza virus, SARS-CoV-2, Middle East Respiratory Syndrome (MERS), RSV and Rhinovirus [41,42]. Adapted from “Epidemiological Comparison of Respiratory Viral Infections”, by BioRender.com (Accessed: 18/02/2022). Retrieved from [43].

Other important biotic agents that most frequently trigger relevant illnesses in humans are respiratory viruses. Common respiratory viruses include influenza virus, coronavirus, respiratory syncytial virus (RSV), and rhinovirus [35]. The sensitivity of the cells to these viruses is characterized by the presence of specific receptors, such as the sialic acid in the cellular membrane that interacts with the virus surface [36]. The same virus may infect a wide variety of organisms, and, depending on the host, the binding site can change, which reflects its high spreading capacity [37]. Moreover, for the species sharing the same receptor, the occurrence of mutations is facilitated since, with increasing diffusion, the probability of genetic modification also increases [37]. Therefore, cell–virus interaction is mediated by surface proteins such as glycoprotein or capsid proteins that define the possible mechanisms for a cell’s occupation (e.g., direct fusion, endocytosis, etc.) [38]. Once into the cell, the virus is able to start the viral genome replication in the host-cell cytoplasm. Based on the diversity of viral action mechanisms, respiratory viruses have distinguished speed and spread rates [37,38,39]. As an example, SARS-CoV-2 can enter human cells through the activation of a spike protein and the binding with the angiotensin-converting enzyme 2 (ACE2); both, the mechanism of activation by spike protein and the high affinity with the binding site, contribute to the improved efficient entry in the cell of SARS-CoV-2 when compared with SARS-CoV [40]. Therefore, some viruses are more able to avoid immune surveillance, which contributes to their quickly becoming widespread [39,40]. Figure 3 compares the most common respiratory viruses in terms of size, binding and replication mechanism, pathology, and control.

For productive infection to be observed, the sensitive cells (which include specific binding sites) should also be permissive to this micro-organism [44]. A non-permissive cell has the respective viral receptor but is not subject to cellular-induced damage resulting from the synthesis and viral replication process [44]. In respiratory-tract viral infections, after the virus is recognized by surface receptors and penetrates the epithelium layer of an epithelial cell, the virus’ genome is released in the cytoplasm or nuclei, breaking their structural membrane [36]. The presence of infiltrating fluids composed of inflammatory cells (neutrophils, lymphocytes, and plasma cells) leads to the inflammatory process with alterations in cell morphology, nuclear modifications and, in some cases, the proliferation of modified cells [44]. The infection of successive cells leads to the spread of the viral genome to the adjacent cellular layers [36,38,44].

The severity of viral infections is dependent on: (i) virus characteristics, such as the incubation time, virulence, spread and replication velocity; (ii) the amount of inoculum; and (iii) the immune defense capacity of the host organism [44]. Some risk factors, such as age, obesity or the prevalence of other comorbidities, have been associated with severe disease caused by viral infection. Usually, prior contact with these pathogenic agents through a controlled and monitored exposure is employed for the enhancement of the immune system’s capacity for severe disease prevention [42]. 

## 3. Electrospun Nanofibers

Electrospun nanofibers result from the jet stretching of a polymeric solution when submitted to an electrostatic force during the electrospinning process [45]. Electrospun meshes have been studied for air-filtering applications since they offer effective respiratory system protection and, consequently, control pathogens spreading [46,47]. In fact, specific electrospun-meshes characteristics, such as reduced fiber diameter, high porosity and porous interconnectivity, provide a barrier that is crucial to physically retaining airborne particles. Therefore, interception, inertial impaction or diffusion play an important role in the barrier effect of fibrillar surfaces [46]. However, the filtration mechanism is not always limited to the physical action of the structure adopted, since the accumulation and proliferation of pathogens in the filter can quickly reduce its effectiveness [48]. Moreover, modified electrospun nanofibers provide a selective response based on chemical reactive surfaces through the incorporation of active agents such as commercial antimicrobial compounds (e.g., quaternary ammonium or phosphonium groups, N-halamine compounds, peptides) or novel metallic NPs, which interact and inactivate potential pathogenic action, namely, silver (Ag), copper oxide (CuO) and magnesium oxide (MgO) [49,50]. 

### 3.1. Physical Barrier

**Figure 4:**
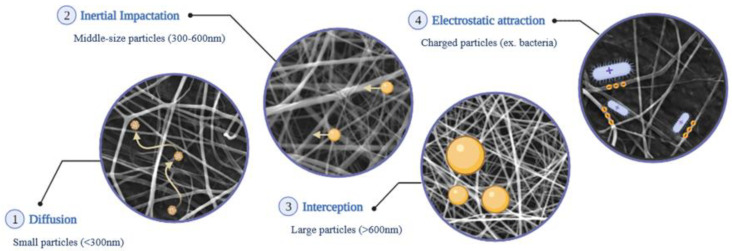
Main filtration mechanisms of fibrillar structures for small (**1**), middle-size (**2**) and large particles (**3**), as well as electrostatic attraction (**4**).

Floating particle size and air velocity are examples of extrinsic parameters that classify the performance of filtration systems [12]. For small particles (<300 nm), filtration can be explained by a diffusion mechanism where the randomness associated with the Brownian motion is responsible for the increased probability of particle–fiber interaction [51,52,53]. The small size of the particles helps during their movement through the pores of the structure and, in this situation, multiple layers are required to maximize the filtration and the probability of particle–fiber interaction [46]. Regarding middle-size particles (300–600 nm), inertial impaction is the mechanism associated with the filtration capacity of membranes [51,52,53]. The dimension of the particle is not enough to overcome the inertial force and maintain the movement after a collision with the fiber; consequently, the particle is stopped [52,53]. For large-sized (>600 nm) particles, the floating particle is easily retained through interception occurring with the reduced-size pores of the mesh. The large size of the particle is a disadvantage for the required change in trajectory when it collides with the fiber and, usually, the particle is retained [12,46]. A visual representation of possible particle–fiber interactions is provided in Figure 4.

The effectiveness of filtration capacity is also related to the intrinsic characteristics of electrospun nanofibers. These characteristics are the orientation of nanofibers adopted, as well as the thickness of the membrane, the size of the pores, and the fibers’ diameter. The random orientation in which nanofibers are deposited in a conventional collector during the electrospinning process is an advantage considering the haphazard trajectory of particles that limit its penetration. Furthermore, electrospun meshes with reduced fiber diameters and high thickness usually result in membranes with improved filtration capacity, since a high volume is required for the particle to cross it. Furthermore, the smaller the size of pores in the membrane is, the higher capacity for particle retention, since it will require bigger pores to pass through [51]. 

Previous studies [54,55,56] evaluated the electrospinning process considering different materials to improve conventional filtering systems. These studies analyzed the influence of polymers and solvent properties in the characteristics of the solution and reported the impact of molecular weight, viscosity, conductivity and polymer concentration on the performance of nanofibers. Therefore, such performance can be affected by the length of molecular chains and the number of chain entanglements, both depending on the molecular weight and the concentration of the polymer [55]. In general, extreme conditions lead to electrospinning jet instability, which may result in heterogeneous diameters, beads and non-continuous fibers [4,56,57]. An undesired morphology can have a high impact on the mechanical and physical properties of electrospun meshes and, consequently, reduce their filtering efficiency and quality factor. The filtering efficiency is calculated based on the ratio of the number of particles at the filter inlet in the airflow direction (upstream) to the number of unfiltered particles (downstream), as shown in the following equation (Equation (1)):

(1) $$E\left(\%\right)=\left[1-\frac{\mathrm{Downstream}\;\mathrm{concentration}}{\mathrm{Upstream}\;\mathrm{concentration}}\right]\times 100\;\;$$

In addition, the quality factor, which depends on the efficiency of the filter, can be obtained through the following equation (Equation (2)):

(2) $$\mathrm{QF}\;\left({\mathrm{Pa}}^{-1}\right)=\frac{-\ln\left(1-E\right)}{\Delta p}\;$$

In Equation (2), 1−E is the ratio of the penetration rate of particles, and ∆p is the pressure drop across the filter. Both efficiency and quality factor have been considered for the standard comparison of a filter’s performance. 

According to the literature, these indicators have shown highly satisfactory results for the synthetic polymers, such as polystyrene (PS), polyacrylonitrile (PAN) or polyimide (PI), that are usually applied in water filters [57,58,59]. The high melting point of these polymers made them also requested for hot-gas filtration systems [58]. Therefore, their electrospinnable capacity was investigated to produce air filters able to operate in high-temperature environments. The promising results reported in these studies showed nanofibers with average diameters of 300 nm and a filtration efficacy above 99% [57,58,59]. However, in the context of air filtering for personal-protection use, most of these polymers are not economically attractive to be considered for industrial propose and other alternatives have been explored. The market-available solutions for air filtering in personal equipment such as surgical masks and protective clothes are usually made from polypropylene (PP) or polyethylene (PE), nonwoven [12]. These polymers are preferred due to their associated mechanical resistance, hydrophobicity, production stability and low cost [60]. However, PP and PE are non-biodegradable materials and their electrospinnable ability is underreported due to their low solubility using common solvents [61]. Alternatives to the conventional polymers used in the development of personal protective air filters are shown in Table 1.

In some situations, the electrospinnability of the polymer has been enhanced by increasing the conductivity of the electrospinning solution [56]. Electrospun nanofibers of polycarbonate (PC) were prepared using as solvents tetrahydrofuran (THF) and dimethylformamide (DMF) [62,63]. Then, hexadecyl trimethyl ammonium bromide (CTAB) was also incorporated to increase the conductivity of the polymeric solution. The produced meshes presented an average fiber diameter of 300 nm and an average thickness of 332 µm and showed a filtering efficiency of more than 95% [62]. The same study compared the efficiency of PS nanofibers with the efficiency obtained for polyvinyl alcohol (PVA) nanofibers [62]. PVA is a biodegradable and environmentally friendly polymer with high electrospinnable capacity [64]. However, PVA nanofibers showed a lower filtering efficacy closer to 75% [62]. In this context, it was proved that Van der Waals forces have an important role in filtering capacity for gas- or liquid-phase molecules, since they explain the adsorption of these molecules by a solid surface [65]. These forces are strongly affected by the materials’ molecular dipole moment; polymers with high dipole moments can provide nanofibers with a higher filtering capacity [62]. Therefore, since PC has a higher dipole moment than PVA, its filtering ability also proved to be higher in this study. 

In addition, polyurethane (PU), which is a biocompatible and non-water-soluble polymer, was investigated to obtain electrospun filters [66]. The nanofibers produced have an average diameter of 140 nm and were tested to filter NPs under 2.5 µm with a proven efficiency of 99.65% [66]. The significant efficiency obtained in this situation shows the relevance of electrospinning for fibrillar-structures production and the relevance of using adequate materials to obtain morphological and mechanically improved membranes. 

In another study, PLA, a strongly hydrophobic and biodegradable polymer, was also reported for electrospun fibers production [67]. Electrospun meshes composed of fibers with diameters under 300 nm were successfully obtained and showed a filtering efficiency closer to 100% when tested for particles with an average size of 250 nm [67]. In addition, polycaprolactone (PCL) was investigated for filtering development also due to its high hydrophobicity and biodegradability [68]. It was verified that, by increasing the deposition time of nanofibers, the pore size could be reduced due to the increased thickness resulting from the overlapping layers [68]. The nanofibers obtained considering a deposition time of 10 min showed a higher angle contact, lower water absorption, higher thickness and, consequently, higher filtration capacity than nanofibers obtained from a deposition time of 3 min [68]. The performance of electrospun nanofibers was compared with the results obtained for traditional surgical masks. The pore size proved to be crucial for particle capture, since their retention was higher for the nanofibers with an average pore size of 1.42 µm than for surgical masks with an average pore size of 5.71 µm [68]. These studies explore the use of environmentally friendly materials for electrospinning production, which is in-line with sustainable product-development guidelines. Other studies also include the use of recycled materials for the development of electrospun nanofibers by using the resultant waste of some industries [69,70]. An industrial transition for the conscience fabrication of novel products and the adoption of better manufacturing practices are encouraged by most governments around the world. 

**Table 1:** Comparison of polymeric nanofibers used for the development of electrospinning-derived air-filtering systems reported in the literature. FD—fiber’s diameter; PM—particulate matter; ∆P—pressure variation; QF—quality factor.

Polymer	Solvent	FD (nm)	E (%)	PM Size (um)	Air Flow Rate	∆P (Pa)	QF (Pa-1)	Ref.
PS	d-limonene:DMF	325	99.99%	*	0.053 m/s	145	0.15	[57]
PI	DMF	300	99.97%	0.3–10	0.2 m/s	73	0.1072	[58]
PC	THF:DMF	~300	>95%	0.3	*	500	*	[62]
	DMF:DCM	90	93.08%	>10	1.5 m3/min	*	*	[63]
PU	DMF:ACTN	140	99.65%	0.25	3.48 m/s	12	*	[66]
PAN	DMF	70–750	99%	0.3	0.042 m/s	27	0.10–0.31	[59]
	DMF	~200	96.12%	0.25	3 m/s	133	0.024	[71]
PVA	Distilled water	~300	98.11%	1–2.5	*	206	0.019	[62]
PLA	DCM:DMAC	273,6	>75%	0.3	5.8 m/s	500	*	[67]

*—data not available.

Previous studies use sodium chloride (NaCl) aerosol generators to create droplet populations in order to evaluate filter performance [72,73]. Then, a particle-size analyzer is used to count and to find the size distribution upstream and downstream and, consequently, to obtain filtering efficiency and quality factor [72,73]. It is important to note that some of the studies in the literature do not consider the standard air velocity, flow and pressure that is observed during human expiratory and inspiratory phenomena [74]. Additionally, the surface area and the duration of the experiment are crucial to defining the shelf-life of electrospun filters, as the time until saturation by fixing the area is considered. For this reason, some of the filtering efficiency values reported required validation for use as personal protective equipment. The lack of an adoption of standard protocols for electrospun-filtering validation has led to significant divergences in the results reported for the same materials with similar characteristics, and makes comparison more difficult during the literature validation process. 

### 3.2. Functional Fibers

While electrospun nanofibers with a small pore size present high filtration efficiency, the pressure drops observed can compromise breathing capacity and, consequently, their use as personal protective equipment [59]. To overcome such issues, other approaches have been considered for the development of equally efficient structures with reduced pressure drop levels. Multilayer systems are one of the most explored filter designs to reduce pressure drop at the same time as filtering efficiency is maintained or improved [51]. Usually, the increased porosity and reduced thickness of each layer compared with the monolayer approach is responsible for promoting higher airflow while offering a physical barrier to other particles [53]. In this situation, the filtering mechanism is still based on barrier methods. However, electrospun nanofibres can be functionalized by incorporating NPs able to chemically interact and inactivate floating aerosols [75,76]. The antibacterial and antiviral activity of electrospun nanofibers after the incorporation of antiseptic drugs, bio-derived polymers or oxide metallic NPs has been reported in the literature [48,77,78]. In addition, the use of oxide metallic NPs proved to enhance filtration efficacy due to the electrostatic attraction imposed by the charged NPs [79].

#### 3.2.1. Biopolymers

**Figure 5:**
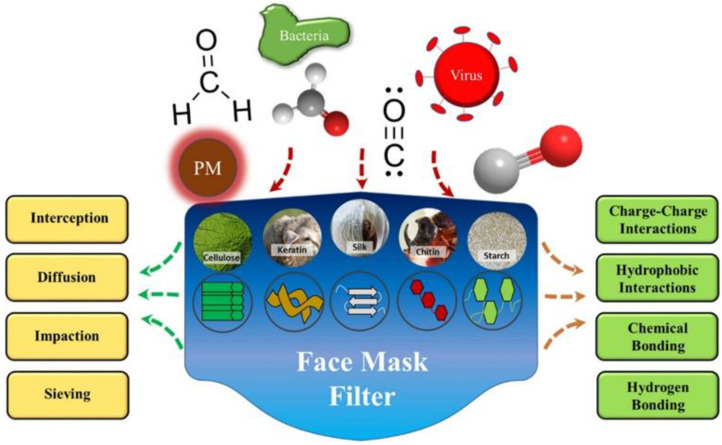
Principal biopolymers used in filtration applications, and their interaction mechanisms with the micro-organisms (virus and bacteria) and other toxic particles. Reproduced with permission from [80].

The application of biopolymers in the biomedical field is extensively reported; however, in recent years, biopolymers have received increasing attention for producing efficient filters, owing to the presence of functional groups, which allow them to possess characteristics such as antibacterial and antiviral properties [80,81,82,83,84,85]. The diverse functional groups that protein- and polysaccharides-based polymers embrace permit them to have different methods of interactions with particles or contaminants [80]. The major biopolymers used for filtering applications are chitosan/chitin, soy protein, silk protein, gelatin, cellulose, keratin, starch, and alginate [80,81,82,83,86] (Figure 5). The principle by which those biopolymers act as filter agents are based on two different approaches: adsorption, where the contaminants are trapped in the filter surface, and the other, chemisorption, where the filter’s components, such as the active sites of biopolymers or metallic particles, react with the contaminant, making them inert. Besides the chemisorption by electrostatic interactions, biopolymers can inert contaminants by inertial impaction, interception, and diffusion as synthetic polymers [80]. 

The main action mechanism of chitosan/chitin against pathogenic organisms, such as bacteria, fungi or mold, are due to their positively charged molecules and negatively charged microbial cell membrane conferring those biopolymers’ antimicrobial activity [80]. Mohraz et al. [86] show that electrospun polyurethane (PU)/chitosan nanofibers reveal antibacterial activity against *E. coli* bacteria as a model micro-organism, and possess other quality factors such as pressure drop, to be used for filtration applications such as industrial filtration processes (air filters) and personal respiratory protection equipment (face masks) [86]. Other authors also developed polymeric composites by combining synthetic and natural materials such as Poly (ethylene terephthalate) (PET) and silk fibroin to achieve the improved performance of electrospun nanofibers in terms of filtration efficiency and mechanical properties, and enhance user comfort. These nanofibers also revealed antibacterial activity against *S. aureus* and *E. coli* bacterium [87].

In recent years, the use of soybean protein in filtration applications has grown considerably as a result of the availability of biomass and the chemical characteristics of this polymer, namely, the presence of 18 different amino acids with many active functional groups capable of interacting with air bone pollutants and pathogenic micro-organisms [81,82]. Soy protein allows an increase in active site for trapping particles or virulent organisms when under acetic conditions, resulting in the deprotonation of acidic and basic groups, such as the carboxyl group, into carboxylate anion (R–COOH to R–COO^-^) and amine groups converting to amino groups (R–NH^3+^ to NH_2_) [81]. In the study of Jiang et al. [84], soy protein isolate (SPI)/polyamide-6 (PA6)-Ag electrospun meshes exhibit high filtration efficiency for both dust particles and toxic gas, and the addition of AgNPs shows great antimicrobial activity against *Escherichia coli* and *Bacillus subtilis*, inhibiting up to 80% of their growth [81]. Cellulose is another biopolymer widely used as biomaterial in several fields, such as biomedical, pharmaceuticals, energy and textile. Cellulose is also suitable for air applications due to its β(1→4) D-glucose units, which contain several hydroxyl groups [82] capable of interacting with several particles, including viruses such as influenza A, HIV-1 or hepatitis A and C and bacteria such as *S. aureus*, *E. coli*, *K. pneumoniae* [80], assuring a high filtration efficiency (>90%) [88].

**Figure 6:**
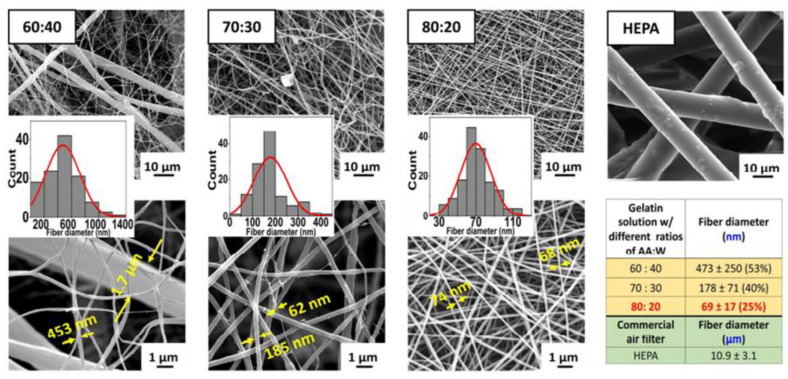
Scanning electron microscopy (SEM) images of gelatin fibers produced with different rations of Acetic Acid-to-water on fibers’ morphology and their filtration capacity, compared to commercial HEPA filter. Reproduced with permission from [90].

Nonetheless, using individually biopolymers in electrospinning is sometimes a demanding task due to their complex structures and viscosity; consequently, several studies use hybrid electrospun meshes, combining natural and synthetic polymers to improve production and performance [17]. For instance, gelatin/β–Cyclodextrin electrospun nanofibers have been shown to adsorb efficiently hazardous VOCs such as xylene, benzene, or formaldehyde [89]. Similarly, the study of Souzandeh et al. reveals that depositing gelatin fibers on paper towel and cellulose-based subtracts enhance significantly the filtration performance of the meshes able to trap small particles (Particule Matter—PM: 0.3) and lower the airflow resistance and pressure drop, respectively, comparing with the commercial filters HEPA (Figure 6) [90]. On the other hand, using only 18 wt% of gelatin as a polymer dissolved in an Acetic Acid-to-water (AA: W) ratio of 80:20, the beadless electrospun fibers efficiently remove PM0.3 and PM2.5 contaminants such as those of the bacteria and viruses, as well as removing chemical gases such as formaldehyde and carbon monoxide [90].

Moreover, keratin is known for excellent hydrophilicity and adsorption properties, allowing the absorption of harmful compounds, but on the other hand, producing fibers of it by electrospinning is quite challenging due to chemical stability and difficult solubilization in most organic solvents [85,89]. Recent studies reveal that keratin electrospun nanofibers combined with nylon had the potential to remove micron-size and suspended solid particles such as flocs and bacteria [91]. Similarly, keratin/polyamide6 nanofibers doped with Ag particles have enhanced filtration and antibacterial performance against *S. aureus* (96.8%) and for *E. coli* (95.6%) [89]. Starch is another biopolymer that shows poor spinnability and, therefore, is usually modified by physical, chemical, and enzymatic modifications or combined with other polymeric materials such as PCL, PVA, PLA, polyethylene oxide, and poly (D, L-lactic-co-glycolic acid) to improve its properties [47]. In the study of Woranuch et al., rice-starch/PVA nanofibers allowed the passage of tiny particles (less than 0.1 micron); however, the incorporation of AgNPs and β-cyclodextrin in the nanofibrous membranes led to excellent antimicrobial properties and VOC-adsorption properties, resulting in a high-performance nanofilter [92]. Moreover, alginate, owing to its gelling capacity, is difficult to use in electrospinning, reducing its application in the air-filtration field [83,85,89]. To overcome this drawback, alginate is combined with several materials, such as chitosan, and nanoparticles. Alginate–Chitosan reveal good microbial filtration against *E. coli* and *S. aureus*; however, adding Ag nanoparticles increases their performance by 1.5 times [83].

The current demand for petroleum-free materials with advantageous properties makes the use of biopolymers in electrospinning a potential and environmentally friendly methodology to apply in air filters.

#### 3.2.2. Inorganic Nanoparticles

The use of metal and metal-oxide NPs to produce electret-stable filters has been explored by incorporating the nanoparticles in polymeric filaments [93]. Electret filters were first produced using polymeric nanofibers which were electrically charged postprocessing to improve filtering efficiency [94,95]. In this situation, the filtration of floating particles is performed through the electrostatic attraction phenomenon due to the difference in charge between the particles and the fibers [96]. However, the short life span of the charged nanofiber, especially in humid environments, led to the search for other alternatives such as the use of electrical reactive NPs [75]. The performance of electrospun nanofibers of polyvinylidene fluoride (PVDF) decorated with silicon dioxide (SiO2) nanoparticles was evaluated to find the enhancement factor due to the electrostatic field produced [73]. As an electret, the PVDF filters showed an efficiency higher than 85%, a pressure drop of approximately 15 Pa and a quality factor (QF) of 0.14 [73]. On the other hand, after the elimination of injected charges, the efficiency was reduced to 51% and the same pressure drop was measured [73]. Similar results are also reported in other studies using inorganic NPs such as TiO2 and Mg, where the charge stored in the electrical material attracts airborne particles and improves filtering efficiency [55,96]. Therefore, inorganic NPs can also contribute to inactivate specific pathogens such as bacteria. Since they were discovered in 1920, antibiotics are preferred as bactericidal or bacteriostatic agents to interrupt the activity of bacteria [75]. However, their application in the filtering industry is limited by product shelf life and drug stability. Several filtering applications have been reported to use metal and metal-oxide NPs to inactivate bacteria [76]. The antibacterial activity of these NPs is due to the electrical interaction with the bacterial cell membrane [79]. The NPs can bind with the cell membrane, change its permeability and release metallic ions into the cell which loses its activity. These ions will promote the generation of reactive oxygen species which can target specific cell components and compromise cell viability [79]. The number of ions released depends on the oxidation susceptibility of elements [75]. In addition, the size, shape and zeta potential of metal and metal-oxide nanoparticles can also influence their capacity to damage Gram-positive and Gram-negative bacteria [75]. Most of the studies report a reduction in bacteria activity using NPs of Ag, gold (Au), zinc oxide (ZnO), CuO, MgO and titanium dioxide (TiO2) [75,76,79]. Some of the studies reporting the antimicrobial activity of inorganic nanoparticles are summarized on Table 2. Several theories have been explored to explain the action of nanoparticles in bacterial cells, since not even all nanoparticles trigger the same events in the different families of bacteria [79]. Electrical interaction between the charged particles and the charges of the cellular membrane has been the most accepted theory to explain particle–bacteria binding [79]. However, this theory does not support all the studies already performed, in which effects of negatively charged nanoparticles in negative bacterial membranes were observed. Therefore, such interaction can result from simultaneous events occurring after the attachment of nanoparticles to cellular-membrane building elements. For most of the inorganic particles, the bacteria inactivation efficiency showed to be improved with the use of smaller nanoparticles, since they have a higher surface area for interaction and can easily penetrate the membrane. Additionally, Gram-positive bacteria seem to be more resistant to nanoparticle-induced damage, probably due to the characteristics of their cellular membrane [76]. For this group of bacteria, the presence of a thicker peptidoglycans layer compared with a Gram-negative cellular membrane may limit ions’ inflow and, consequently, the concentration of harmful reactive oxygen species in cytoplasm [79].

**Table 2:** Electrospun nanofibers containing inorganic nanoparticles used for the development of air filters reported in literature. FD—fiber’s diameter; NP—nanoparticle type; NP Size—nanoparticle size; PM—particulate matter; E—filtration efficiency; ∆P—pressure variation; QF—quality factor.

Polymer	NP	NP Size (nm)	Activity	FD (nm)	PM Size (um)	E (%)	Air Flow Rate	∆P (Pa)	QF (Pa^−1^)	Ref
PU	CuO	50–1000	*E. coli*	182–226	*	> 95	*	*	*	[97]
			*S. gallinarum*							
PAN	ZnO	*	*E. coli*	200–300	*	99.91	30–160 L/min	200–900	*	[50]
			*S. aureus*							
PAN	Ag	<50	*E. coli*	250–400	*	~ 100	0.3–3 cm/s	200	>0.04	[72]
PAN/PVDF	Ag	1–10	*S. aureus*	~171	0.3	~95	32 L/min	168	~0.07	[98]
			*K. pneumonia*							
			*M. smegmatis*							
			*M. tuberculosis*							
			*H1N1*							
PA6	Ag	*	*E. coli*	90	2.5	99.99	32 L/min	31	0.3	[48]
			*S. aureus*							
			*P. detalcoronavirus*							

*—data not available.

**Figure 7:**
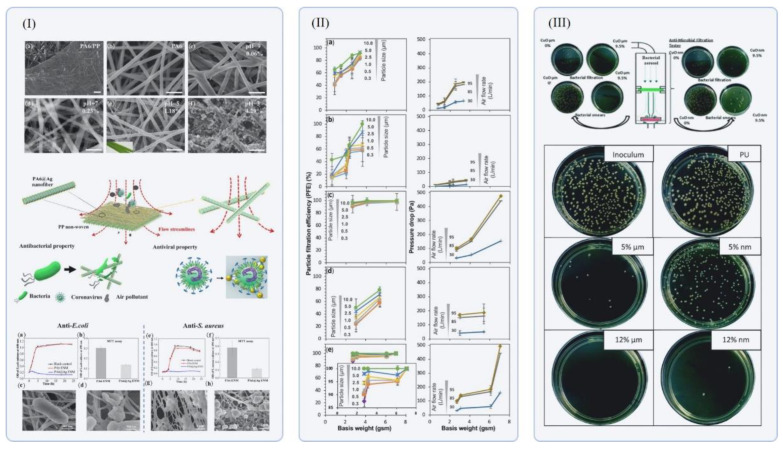
(I) Schematics showing antibacterial activity against *E. coli* (**a**,**b**) and the morphology (**c**,**d**) of these bacteria on PA6 electrospun nanofibers without and with Ag NPs, respectively; as well as the results obtained regarding their antibacterial activity against *S. aureus* (**e**,**f**) and the morphology (**c**,**d**) of these bacteria on PA6 nanofibers without and with Ag NPs (**g**,**h**), respectively. Adapted with permission from [48]. (**II**) Evaluation of electrospun nanofibers on particle filtration efficiency of (**a**) PAN, (**b**) PAN/PVDF, (**c**) PAN/PVDF/GM, (**d**) PAN/PVDF/Ag and (**e**) PAN/PVDF/GM/Ag. Adapted with permission from [98]. (**III**) Schematic representation of antibacterial activity evaluation of polyurethane nanofibers incorporating CuO NPs against Staphylococcus gallinarum. Adapted with permission from [97].

Previous studies [83,98] with Ag NPs (1–10 nm) have demonstrated high efficiency in the inactivation of a large range of micro-organisms, namely, *S. aureus*, *K. pneumonia*, *M. smegmatis* and *M. tuberculosis* bacteria, but also in the inactivation of the H1N1 virus. In this situation, a study developed by Saikaew et al. demonstrated that PAN/PVDF nanofiber physical-filtration efficiency was not affected by the incorporation of Ag nanoparticles and it was maintained at 95% [98]. In addition, *E. coli* and *P. detalcoronavirus* proved to be sensitive to Ag nanoparticles when they were incorporated in PA6 nanofibers [48]. These results are shown in Figure 6, where it is possible to observe a significant decrease in bacterial-growth proliferation rate. In addition, nanoparticles of CuO and ZnO have been shown to have antibacterial activity against Gram-positive and Gram-negative bacteria with filtering efficiencies higher than 95 and 99%, respectively, as also shown in Figure 7 [50,97]. In terms of morphology, the addition of metallic nanoparticles into the electrospun fibers induces a jet stretching due to the increase in solution conductivity resulting in thinner fiber diameters [50]. Therefore, nanofibers with lower diameters contributed to the reduction in pressure drop while the presence of metal and metal-oxide NPs improved the filtration efficiency and antimicrobial activity of these membranes [79]. Several concerns have been discussed in terms of the biodegradability of electrospun nanofibers containing metal oxides. Depending on the metal oxide and the polymer used during the electrospinning process to encapsulate the nanoparticles, the electrospun mesh can be recyclable under low temperatures. Optimizing the recycling process of masks containing metal nanoparticles such as copper oxide may be a crucial step to avoid severe impact on marine organisms [99]. However, other inorganic nanoparticles, such as, for example, magnesium oxide, present a more sustainable option. Inorganic nanoparticles presenting a reduce risk for terrestrial and marine environment, but also having significant filtering performance and electrospinnability, should be considered and preferred [46,99].

## 4. Commercial Electrospun Face Masks

Nowadays, the use of face masks is a standard safety procedure that has been added to daily routines. The most common masks used are face/surgical masks and the respirator masks FFP2/N95. The first ones are designed for light medical settings and do not necessarily protect the wearer from bacteria and viruses and the second ones are specially designed for high-risk medical settings and can protect the wearer from different pathogens [100].

As mentioned above, the main components of the protective masks on the market are mainly melt-blown nonwoven, which are made of PP and can adsorb and filter fine dust with a diameter of less than 0.3 µm through electrostatic mechanisms. This static electricity is easily lost, especially after washing or wearing for a long time, which makes these masks disposable [51]. This, in association with the fast development of nanotechnology during the 21st century as well as the needs caused by the current pandemic situation, led to the production of high-efficiency nanofibrous materials for air-filter applications. Therefore, more and more research groups have put their efforts into studying electrospinning to produce new functional nanofiber membranes that are reusable, cleanable, and degradable [101]. Electrospinning, with the advantages of being simple equipment, having a controllable small diameter, its porous structure, a high surface-area-to-volume ratio, good internal connectivity, and controllable morphology guarantee excellent filtering performance and controllable filtration at low cost, meaning it has become the preferred method for the preparation of polymer nanofibers [51]. In fact, the nanofiber market is growing so fast that is now valued at ~EUR 685 million and is expected to register a compound annual growth rate (CAGR) of around 17.5% during the forecast period (2021–2026) (and, a large piece of this amount is related to products to fight SARS-Cov-2, namely, masks and filters) [102].

Concerning all the advantages considered and the current world situation, it is clear that this technology has the potential to be translated to the market (Table 3). In fact, this has already happened, with several models being advertised by companies as advanced last-generation filters/masks with multiple properties that can go from unique filtration/permeability properties to antimicrobial effects.

One example of these companies is AIRQUEEN (Seoul, Korea) which combines polyurethane and electrospinning to produce masks based on nanomembranes carefully designed to produce 3D-web structures. Thus, the nanomembrane provides outstanding airflow without compromising waterproofness [103]. Another example is from Protek Nano company, namely, the Protek Nano P2 mask, which additionally filters different kind of particles efficiently, and also kills 99% of viruses that make contact with its surface. Due to these properties, this mask also minimizes potential biohazard risks after its disposal [104]. Below is presented some examples of commercial masks produced using the electrospinning technique and their main characteristics:

**Table 3:** Commercial masks produced using electrospinning technique.

Commercial Product Name/ Company	Material	Main Properties	Unit Cost (EUR)	Country	Ref.
YAMASHIN Nano Filter™	n.p.	Extremely thin fibers with less than one-tenth of general synthetic fibers containing super-high trapping properties. Tests have shown almost no decrease in collection performance even after long-term use (no dependence on static electricity).	-	Japan	[105]
AIRQUEENnano mask	Polyurethane	Blocks a minimum of 95% of particles while allowing superior air flow to enable outstanding breathability (independent testing shows an average of +97% filtration).	4.22	USA	[103]
E-Spin Nanotech Pvt Ltd.	n.p.	The facemasks have 99.9% filtration efficiency with low pressure drop and enable any kind of lethal virus penetration below 100 nm size.	-	India	[106]
NASK nanofiber respirator	n.p.	Present more than 99% filtration efficiency against most penetrating particles. In addition, present more than 99% bacterial and viral filtration efficiency. Additionally, the nanofibers have a bactericidal effect capable of killing 99% content of bacteria within 5 min.	2.06	Japan	[107]
FNM RespiNano mask	n.p.	Respiratory mask with an efficiency of 94% (for 0.3 µm particles) and pressure drop of 61 Pa suitable for hospitals and mine contaminations with non-toxic chemicals.	-	Iran	[108]
RespiRaptor	PVDF	Captures up to 99.9% of viruses and also captures bacteria, smog, dust, pollen, allergens, mold spores and other pathogens. Contrary to ordinary respirators, the level of filtration efficiency of this nanorespirator stays the same regardless of air humidity (and of the humid human breath as well).	2.75	Czech Republic	[109,110]
Proveil^®^ FFP2	n.p.	Extremely thin and lightweight with a pore size more than 10 times smaller than conventional materials leading to mechanical filtration without reliance on electrostatics. The filter performance is maintained over time or when exposed to humidity. Is based on a sandwich of spunbond/ nanofiber/spunbond materials.	-	Spain	[111]
K-MASK	51% KYnergy Polyester 35% Polyester 14% Spandex	Protect from viruses, bacteria and environmental particulates, with a 99.6%. Use a triple sandwich layer nanofibre combination: layer 1—nanofibres capture viruses, bacteria and pollutants via electrostatic attraction; layer 2—activated carbon layer traps environmental pollutants; layer 3—nanofibres capture viruses, bacteria and pollutants via electrostatic attraction.	36.95	UK	[112]
Protek Nano P2	n.p.	This antiviral respirator kills 99% of viruses and can be effectively worn for up to 24 h before disposal. Additionally, present four layers, leading it to capture both large droplets and tiny airborne particles. Since this filter deactivates and kills the virus, it protects the wearer and those around them and minimizes potential biohazard risks after disposal.	2.85	Australia	[104]
FilterLayr™ Eco	patent-pending formulation containing natural manuka oil	Bactericidal and virucidal properties for air-filtration products and highly efficient for PM protection with 99.99% of particles trapped.	-	New Zeland	[113]
INOFILTER V filtration media	PET and PVDF	This mask presents low breathing resistance and protection from airborne bacteria and viruses (99.9% viral filtration efficiency). Additionally, it blocks viruses and bacteria during inhalation and also exhalation. Has resistance against liquids such as blood and oil, among others, and presents highly efficient mechanical filtration.	-	Turkey	[114]

n.p—not provided.

Following these already-on-the-market-electrospun-based masks, the research and the patents in the field have increased over the last few years, which leads us to predict that the number of products made by this technology tends to increase exponentially. Overall, nanotechnology is playing a crucial role in the air-filtration field, as the nano-based masks produced are endowed with the ability to not lose their efficiency with time (because of their mechanical filtration efficiency protection due to the mask layers).

## 5. Standards for Filtering Development

The International Organization for Standardization (ISO) and European Standards (EN/EEC) guide the development and certification process of personal protective equipment (PPE); the ones concerning caps, gowns, full protective suits or masks are highlighted in the work [115]. Beyond that, PPE Directive 89/686/EEC is responsible for harmonizing procedures related to PPE development in the European economic area [115]. In this context, several standard guidelines were developed under this directive, including EN 14126:2003, EN 14605:2009, EN 13795-1:2019, EN 13795-2:2019 for caps, gowns and full protective suits; as well as EN 149:2009, EN 14683:2019 for face masks [116,117,118,119]. In addition, international standards guide PPE development, namely, ISO/TC 94/SC 13 which is recognized globally for regulation of protective cloths [120]. Other institutions as the American Society for Testing and Materials (ASTM) also contribute to the development of evaluation methods, classification, and terminology standards applied in the research field for product validation [121,122,123]. In general, barrier protective textile, masks and respirators are submitted to several tests imposed by governments to evaluate compliance with the mechanical properties, antimicrobial activity and filtration performance required. Therefore, mechanical and physical tests include the evaluation of tensile strength and elongation of materials (ISO 29073-3:1993), as well as their resistance to hydrostatic pressure (ISO 811:2018, ASTM F1862), industrial washing (ISO 6330:2012) and evaluation of the air permeability of membranes (9237:1995) to ensure the appropriate breathability for face-mask application [121,124,125]. In addition, recommended tests for analysis of filtration capacity consider the bacterial-filtration efficiency (ISO 22610:2006, ASTM F2101) and particle-holding capacity (ASTM F2299) [122,123,126]. Furthermore, for more sophisticated filtering systems, the antimicrobial activity of materials has been tested (ISO 20743:2013) in order to prove their ability to limit micro-organism spread [127].

## 6. Conclusions

The pandemic demonstrated clearly how vulnerable the worldwide population is to respiratory infectious agents; consequently, the use of face masks became crucial to control and eliminate their spread. However, the global demand for face masks in the pandemic proved the need for alternatives to the traditional nonwoven production techniques. Based on this, the electrospinning technique has been established as a real alternative due to its ability to develop meshes with small pores and bioactive fibers. Thus, electrospinning meshes, beyond increasing filtering efficiency, can also eliminate pathogenic agents through the incorporation of bioactive mediators. There are some electrospinning masks available on the market; most of them use single materials and present undifferentiated layers. Nevertheless, all demonstrated high filtering efficiency and some present the ability to kill the pathogenic agents already. Based on these, the structural and chemical characteristics of electrospun-based membranes provide a key tool to prevent respiratory infections. Further studies should focus on the development of more sustainable alternatives by using recyclable, biodegradable and self-cleaning materials with the aim of reducing the ecological footprint associated with face masks.

## Data Availability

The raw data required to reproduce these findings cannot be shared at this time as the data also form part of an ongoing study.
